# High prevalence and genetic diversity of hemoplasmas in bats and bat ectoparasites from China

**DOI:** 10.1016/j.onehlt.2023.100498

**Published:** 2023-02-06

**Authors:** Rui Wang, Ze-Min Li, Qiu-Ming Peng, Xiao-Lan Gu, Chuan-Min Zhou, Xiao Xiao, Hui-Ju Han, Xue-Jie Yu

**Affiliations:** a State Key Laboratory of Virology, School of Public Health, Wuhan University, Wuhan, Hubei, China; b Institute of Epidemiology Research, Hubei University of Chinese Medicine, Wuhan, Hubei, China; c School of Public Health, Shandong First Medical University & Shandong, Academy of Medical Sciences, Ji'nan, Shandong, China

**Keywords:** Bat, Ectoparasites, Hemoplasmas, Hemotropic mycoplasmas, Genetic diversity

## Abstract

Hemoplasmas can cause severe hemolytic anemia in humans. To explore the genetic diversity and the potential transmission routes of hemoplasmas among bat population, bats and bat-ectoparasites including bat-flies, bat-mites, and bat-ticks were collected in Eastern and Central China from 2015 to 2021, and tested with PCR for hemoplasmas 16S rRNA gene. Based on 16S rRNA PCR, 18.0% (103/572) adult bats were positive for hemoplasmas, but none of 11 fetuses from hemoplasmas-positive pregnant bats was positive for hemoplasmas. These results indicated that adult bats had a high prevalence of hemoplasma, but vertical transmission of hemoplasmas did not occurr in the bats. Based on the 16S rRNA gene PCR, the minimum infection rate of bat-ectoparasite for hemoplasmas was 4.0% (27/676), suggesting that bat-ectoparasite also had a high prevalence for hemoplasmas. Phylogenetic analysis revealed that bat hemoplasmas from this study clustered into 4 genotypes (I-IV). Genotype I clustered together with hemoplasmas identified in bats from America. Genotype II shared high similarity with a human-pathogenic hemoplasma *Candidatus* Mycoplasma haemohominis. Genotype III and IV were unique, representing 2 new hemoplasma genotypes. Only genotype I was identified in both bats and all bat-ectoparasites including bat-flies, bat-mites, and bat-ticks. In conclusion, bats and bat-ectoparasites from China harbored abundant genetically diverse hemoplasmas including potential human-pathogenic hemoplasmas, indicating bats and bat-ectoparasites may play important roles in the maintenance and transmission of hemoplasmas in the natural foci.

## Highlights

• Bats from China carry a diversity of novel hemoplasmas with a high prevalence, including potential human-pathogens.
• Bat-ectoparasites including fly, mite, and tick also carry hemoplasmas and they may be the vectors of hemoplasmas in bats.

## 1 Introduction

Bats belong to the order Chiroptera, which is the second largest order of mammals next to Rodentia, and are geographically widespread, especially abundant in the tropical and subtropical regions. As the only mammal that develop the true capability of powered flight, bats can travel a long distance, and have a wide range of activities. Bats are important sources of emerging infectious diseases, and they are considered as natural reservoirs of several viruses that cause severe diseases in humans, such as Hendra virus, Nipah virus, Marburg virus, MERS coronavirus, SARS coronavirus [1,2]. During the past two decades, a large number of novel viruses have been identified in bats worldwide [3], while bacterial pathogens in bats remain much more neglected [4].

**Fig. 1:**
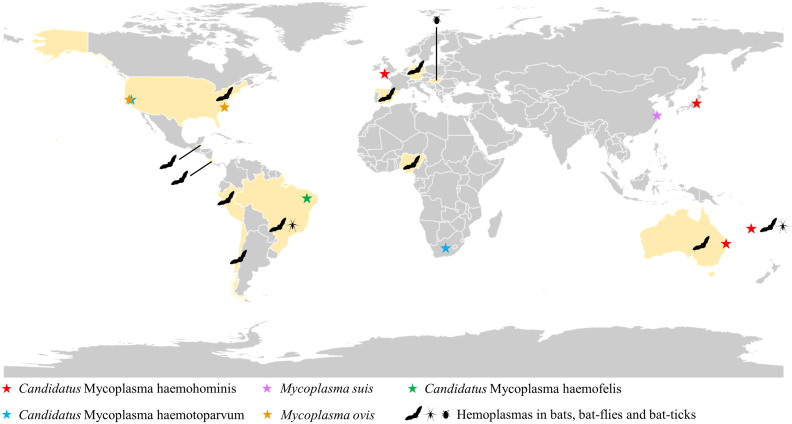
Map showing current knowledge on human infection with epierythrocytic mycoplasmas and bat-related hemoplasmas. Countries or regions that reported human cases were marked with the pentagram (★). Countries or regions that reported hemoplasmas in bats and bat-ectoparasites were highlighted and marked with icons (bat, bat-fly, bat-tick).

Hemoplasmas, also known as hemotropic mycoplasmas, are epierythrocytic parasites within the order Mycoplasmatales, which were formerly known as *Haemobartonella* and *Eperythrozoon.* They are unculturable, cell wall-less bacteria that parasitize on the surface of erythrocytes of diverse mammals [5]. Hemoplasmas infections in humans and animals vary from asymptomatic to severe hemolytic anemia [6]. A diversity of hemoplasmas was identified in a wide range of wild and domestic animals [[7], [8], [9], [10]]. Several hemoplasmas were reported to cause human infection, including *Candidatus* Mycoplasma haematoparvum from dog, *M. ovis* from sheep, *M. haemofelis* from cat, and *M. suis* from pig [[11], [12], [13], [14]] (Fig. 1).

A diversity of novel hemoplasmas was identified in bats from several countries, mostly in the Americas (the United States, Chile, Belize, Brazil, Peru, and Costa Rica), also in Australia, New Caledonia, Nigeria, Germany, and Spain [[15], [16], [17], [18], [19], [20], [21], [22], [23]] (Fig. 1). However, there is a lack of understanding on the genetic diversity of hemoplasmas in bats from Asia. Cases of human infection of *Candidatus* Mycoplasma haemohominis, which usually leads to severe febrile splenomegaly and autoimmune hemolytic anemia, even spleen rupture with fatal outcomes, have been reported world widely including the United Kingdom, Japan, New Caledonia, and Australia [16,[24], [25], [26]] in recent years (Fig. 1). Thus, *Ca*. M. haemohominis is likely an emerging disease in humans, and that evidence in New Caledonia [16] suggests zoonotic potential from bats. Currently, it is unknown how hemoplasmas are maintained and transmitted among bats. Although researchers suggested that hemoplasmas in bats appear to be transmitted through direct contact and arthropods [6], more evidence is in need to clarify the transmission mode of hemoplasmas among bat population.

In this study, the prevalence and genetic diversity of hemoplasmas in bats and bat-ectoparasites from Eastern and Central China was investigated. Besides, the potential roles of vector-borne transmission and vertical transmission of hemoplasmas among bats were evaluated.

## 2 Materials and methods

### 2.1 Ethics statement

The bat capture for microbiological studies was approved by the Ethics Committee of the Medical School, Wuhan University (WHU2020-YF0023).

### 2.2 Sampling and species identification of bats and bats ectoparasites

Bats and bat ectoparasites were sampled during an ongoing program of identifying pathogens in bats. Three rural areas were selected for sample collection sites including 1 site from a prefectural city (Linyi city, 117°51′58.60“ E / 35°42’10.58” N) in Shandong Province in Eastern China, 2 areas from 2 prefectural cities (Xianning city, 114°18′50.63“ E / 29°47’5.83” N, and Jingzhou city 111°51′3.25“ E / 30°7’44.85” N) in Hubei Province in Central China. Mist nets were settled near the entrance of karst caves before sunset. Once captured, bats were sacrificed by inhaling ethyl ether, and transported to the laboratories on ice immediately. Information about bat species, sex, and reproductive status was recorded. Bat-ectoparasites were collected from the wing and fur of bats using forceps. Bat blood and organ samples were collected and stored at −80 °C before analysis. Bats and ectoparasites were identified by taxonomic morphological keys [[27], [28], [29], [30]], and 3–5 samples of each species were subjected to PCR assay targeting mitochondrial cytochrome B (*cytB*) gene, mitochondrial cytochrome oxidase subunit I (*COI*) or 16S rRNA gene to confirm the accuracy of the classification [[31], [32], [33]] (Table S1). For bat samples lack of morphological information, species were identified by amplifying and sequencing *cytB* gene as described above.

### 2.3 Molecular detection of hemoplasmas in bats and bat-ectoparasites

DNA was extracted from bat blood or liver samples, bat-flies, bat-mites, and bat-ticks using Trelief Animal Genomic DNA Kit (Tsingke, Beijing, China). All the DNA samples were stored at −20 °C before analysis.

For hemoplasma detection, a conventional PCR targeting a 600 bp fragment of the 16S rRNA was performed as previously described [34] (Table S1) for initial screening. PCR was carried out under the following conditions: an initial denaturing cycle at 95 °C for 5min followed by 35 cycles at 95 °C for 30 s, 65 °C for 30 s, and 72 °C for 60 s, and a final extension cycle at 72 °C for 10min. Negative controls were set in each PCR assay using nuclease-free water instead of DNA as PCR template. To avoid cross-contamination, no positive control was included. PCR products were analyzed by electrophoresis on 1.2% agarose gel stained with Goldview™ Nucleic Acid Gel Stain (10,000×) (GL biotech, Shanghai, China). Positive samples were additionally subjected to PCR assays targeting a longer fragment of the 16S rRNA gene, and a 300 bp fragment of the 23S rRNA gene of hemoplasmas (Table S1). Agarose gel bands with expected size were excised, and extracted with SanPrep Column DNA Gel Extraction Kit (Sangon Biotech, Shanghai, China). Purified PCR products were directly sequenced, or inserted into T-Vector pMD19 (Simple) (Takara, Shiga, Japan) for TA cloning. At least three positive colonies of each sample were sequenced with universal primers M13–47 and M13–48.

### 2.4 Phylogenetic analysis

Chromatograms were checked with Chromas 2.5.1 (Technelysium, Tewantin, QLD, Australia) to ensure the accuracy of sequencing. Qualified sequences were then compared to the existing sequences deposited in GenBank with Basic Local Alignment Search Tool (BLAST) on National Center for Biotechnology Information (NCBI). Sequences from this study and reference sequences downloaded from the GenBank database were imported into MEGA-X (https://www.megasoftware.net), and aligned with Cluster W. Best-fit evolutionary model was chosen following Bayesian Information Criterion (BIC) to build phylogenetic trees, and *Mycoplasma pneumoniae* was used as outgroup. A haplotype data file of 16S rRNA was generated in DnaSP 5 (http://www.ub.edu/dnasp/) using aligned sequences, and a nucleotide sequence type (ntST) network was then conducted in PopArt (http://popart.otago.ac.nz/index.shtml) with Median Joining network method [35].

### 2.5 Statistical analyses

The positive rate of hemoplasmas by sample type was analyzed using Chi-square test. Information of sex, and reproductive status (pregnant or not) was only collected in samples in 2021, and details of ectoparasite index was not recorded. Hence, in order to evaluate the prevalence of hemoplasmas by these factors, Chi-square test or Fisher's exact test was performed using available data in 2021 with SPSS 21.0 (https://www.ibm.com/analytics/spss-statistics-software), and differences were statistically significant if *P* values were <0.05.

## 3 Results

### 3.1 Sampling and species identification of bats and bat-ectoparasites

In total, 608 bats including 572 adults, and 36 fetuses were collected from rural areas in three prefectural cities (Linyi, Xianning, Jingzhou) in Shandong, and Hubei provinces from 2015 to 2021. The bats were classified into 12 species of 3 families, including *Miniopterus schreibersii* in the family of Miniopteridae, *Hypsugo alaschanicus*, *Eptesicus serotinus*, *Myotis adversus*, *Myotis altarium*, *Myotis davidii*, *Myotis fimbriatus*, *Myotis laniger*, *Myotis pequinius*, and *Myotis ricketti* in the family of Vespertilionidae, *Rhinolophus pusillus*, and *Rhinolophus ferrumequinum* in the family of Rhinolophidae (Table 1).

**Table 1:** Sampling information and prevalence of hemoplasmas in bats from Shandong and Hubei provinces, China from 2015 to 2021.

Sampling time	Sampling area	Host animal	Sample type	No. of samples	No. of positive samples (%)
March – October 2015	Mengyin District, Linyi, Shandong	Rhinolophidae			
		*R. ferrumequinum*	blood	2	0
		Vespertilionidae			
		*E. serotinus*	blood	23	1 (4.3)
		*M. ricketti*		5	1 (20.0)
		*M. fimbriatus*		11	5 (45.5)
		*M. penquinius*		48	0
Subtotal				89	7 (7.9)
May 2018	Xianan District, Xianning, Hubei	Miniopteridae			
		*M. schreibersii*	liver	8	0
		Vespertilionidae			
		*H. alaschanicus*	liver	4	0
		*E. serotinus*		2	0
		*M. adversus*		28	0
		*M. altarium*		2	0
		*M. davidii*		36	1 (2.7)
		*M. laniger*		2	0
		*M. fimbriatus*		3	0
		*M. pequinius*		53	0
		*M. ricketti*		2	0
Subtotal				140	1 (0.7%)
September 2019	Songzi District, Jingzhou, Hubei	Miniopteridae			
		*M. schreibersii*	liver	3	0
		Vespertilionidae			
		*M. davidii*	liver	13	7 (53.8)
Subtotal				16	7 (43.8)
July 2020	Tongshan County, Xianning, Hubei	Vespertilionidae			
		*M. adversus*	liver	24	1 (4.2)
		*M. laniger*		1	0
		*M. davidii*		87	1 (1.1)
			blood	15	0
		Rhinolophidae			
		*R. pusillus*	liver	56	4 (7.1)
			blood	18	2 (11.1)
Subtotal				201	8 (4.0)
May 2021	Tongshan County, Xianning, Hubei	Miniopteridae			
		*M. schreibersii*	blood	4 _(1)_	4 (80)
			liver	2 _(2)_	0
		Vespertilionidae			
		*M. adversus*	blood	47 _(1)_	40 (85.1)
		*M. davidii*	blood	48 _(7)_	34 (70.8)
			liver	25 _(25)_	2 (8)
Subtotal				126 _(36)_	80 (64.0)
**Total**				**572**_**(36)**_	**103 (18.0)**

Note: Subscripts in the parentheses represented the number of pregnant bats.

Abbreviations: *R. ferrumequinum*, *Rhinolophus ferrumequinum*; *E. serotinus*, *Eptesicus serotinus*; *M. ricketti*, *Myotis ricketti*; *M. fimbriatus*, *Myotis fimbriatus*; *M. penquinius*, *Myotis pequinius*; *M. schreibersii*, *Miniopterus schreibersii*; *H. alaschanicus, Hypsugo alaschanicus*; *M. adversus*, *Myotis adversus*; *M. altarium*, *Myotis altarium*; *M. davidii*, *Myotis davidii*; *M. laniger*, *Myotis laniger*; *R. pusillus, Rhinolophus pusillus.*

A total of 676 bat-ectoparasites including bat-flies, bat-mites, and bat-ticks were retrieved from bats collected in Tongshan, Xianning City, Hubei Province from 2020 to 2021. Based on the taxonomic morphological keys, bat-flies were classified into two species: the larger bat-flies (*Penicillidia monoceros*), and the smaller bat-flies (*Nycteribia* sp.). Bat-mites were classified into *Spinturnix* sp. and *Eyndhovenia* sp., and all bat-ticks were identified as *Ixodes simplex*. The larger bat-flies and adult bat-ticks were individually processed, while the smaller bat-flies, bat-mites, and bat-tick nymphs were pooled for DNA extraction (2–20 individuals per pool). Species identification of bat-ectoparasites collected in 2020 were described previously [36]. Together, 300 DNA samples were obtained from bat-ectoparasites (Table 2).

**Table 2:** Sampling information and prevalence of hemoplasmas in bat ectoparasites (bat-flies, bat-mites, and bat-ticks) from Hubei Province, China from 2020 to 2021.

Sampling time	Sampling area	Ectoparasites	No. of samples	No. of DNA samples	No. of positive samples	Minimum infection rate (%)
July 2020	Tongshan County, Xianning City, Hubei Province	Nycteribiidae				
		*P. monoceros*	119	119	11	9.2
		Spinturnicidae				
		*Spinturnix* sp.	200	11‡	1	0.5
		*Eyndhovenia* sp.				
Subtotal			319	130	12	3.8
May 2021	Tongshan County, Xianning City, Hubei Province	Nycteribiidae				
		*P. monoceros*	88	88	7	8.0
		*Nycteribia* sp.	40	13‡	1	2.5
		Spinturnicidae				
		*Spinturnix* sp.	140	22‡	4	2.9
		*Eyndhovenia* sp.				
		Ixodidae				
		*I. simplex*	26	26	2	7.7
			63	21‡	1	1.6
Subtotal			357	170	15	4.2
**Total**			**676**	**300**	**27**	4.0

Abbreviations: *P. monoceros*, *Penicillidia monoceros*; *I. simplex*, *Ixodes simplex*.

‡ Samples in pools.

Representative sequences of bat species (*cytB*) and bat-ectoparasites (16S rRNA, *COI*) were deposited in the GenBank with accession numbers KX655809-KX655810, KX655817, KX655826, KX655831, KX655837, KX655840, MH888177-MH888183, MH888178, MW085077-MW085079, OP779865-OP779887 (bat), OP758801-OP758805 (bat-fly), MZ483874- MZ483876, MZ238086- MZ238089 (bat-mite), OP750378-OP750380 (bat-tick).

### 3.2 Prevalence of hemoplasmas in bats and bat-ectoparasites

The 16S rRNA PCR showed that 18.0% (103/572) bats were hemoplasma positive. Seven out of 12 bat species were positive for hemoplasmas, including *M. schreibersii* in the family of Miniopteridae, *E. serotinus*, *M. adversus*, *M. davidii*, *M. fimbriatus*, and *M. ricketti* in the family of Vespertilionidae, and *R*. *pusilus* in the family of Rhinolophidae (Table 1). For bats sampled during the breeding season, 31.4% (11/36) pregnant bats were hemoplasma-positive, but their fetuses were all negative to hemoplasmas. No statistical difference was observed between the positive rate among bat sexes (*P* = 0.39 > 0.05), or female bats with different reproductive status (*P* = 1.00 > 0.05).

We obtained blood, liver, and spleen from each bat collected from Tongshan, Hubei Province in 2021. Thus, we determined the hemoplasma positive rate in different bat sample types. The 16S rRNA PCR showed that the positive rate in blood, liver, and spleen were 78.8% (78/99), 21.7% (20/92), and 2.3% (2/88), respectively. The hemoplasma infection rate is significantly high in blood sample than in livers and spleen tissues (*χ2* = 130.5, *P* < 0.001, Table S2).

The hemoplasma positive rate in bat-ectoparasites collected from Tongshan, Xianning, Hubei was 4.0% (27/676). For bat-flies, the hemoplasma positive rate in the larger bat-flies (*P. monoceros*) collected in 2020 and 2021 were 9.2% (11/119) and 8.0% (7/88), respectively; and for the smaller bat-flies (*Nycteribia* sp.) collected in 2021, the minimum infection rate (MIR) was 2.5% (1/40). The hemoplasma MIR for bat-mites (S*pinturnix* sp., and *Eyndhovenia* sp.) collected in 2020 and 2021 were 0.5% (1/200) and 2.9% (4/140), respectively. For bat-ticks (*I. simplex*) collected in 2021, the hemoplasma positive rate was 7.7% (2/26) for adult ticks, and 1.6% (1/63) for nymph ticks, respectively (Table 2). Statistical difference in hemoplasma positive rate was observed between larger bat-flies and bat-mites in 2020 (*χ2* = 13.4, *P* < 0.001).

### 3.3 Phylogenetic analysis

**Fig. 2:**
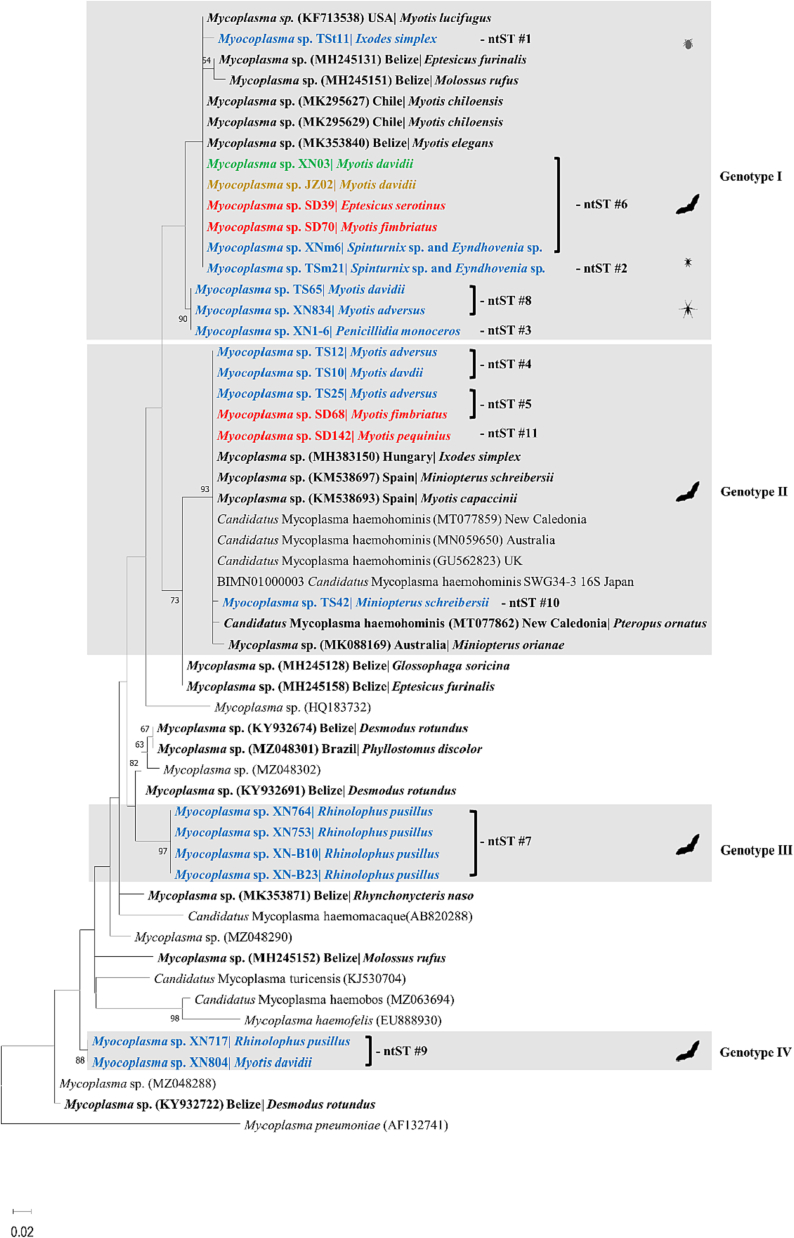
Phylogenetic analysis of hemoplasmas based on 16S rRNA gene (approximately 562 bp). The phylogenetic tree was constructed using the Maximum-likelihood method, Kimura 2-parameter model with Gamma distribution (K2 + G) and 1000 bootstrap analysis in MEGA**-**X. Sequences from this study were color-coded and sequences obtained from bats and bat-ectoparasites were shown in bold. Genotypes detected in this study were highlighted in grey boxes.

**Fig. 3:**
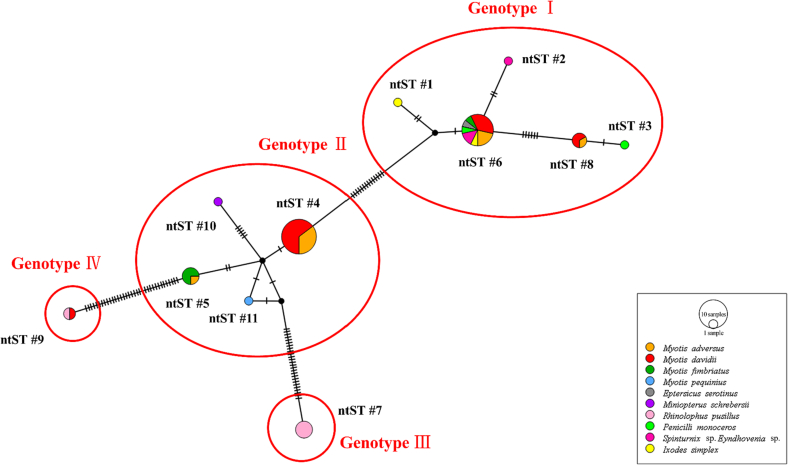
Nucleotide sequence type (ntST) network with hemoplasma 16S rRNA sequences (approximately 535 bp). Each pie chart represented a different ntST, and pie size was scaled by the ntST frequency, while different pie color corresponding to different sample species. Genotypes defined by phylogenetic analysis were indicated in red circles. (For interpretation of the references to color in this figure legend, the reader is referred to the web version of this article.)

Of the 130 hemoplasma positive samples, 16S rRNA gene sequences were obtained from 85 samples with intense PCR bands. Based on the phylogenetic analysis of the 16S rRNA gene, hemoplasmas identified in bats and their ectoparasites were divided into four genotypes (I-IV), among them 2 hemoplasma strains might represent novel genotypes of hemoplasmas (III-IV) (Fig. 3). Eleven ntSTs of hemoplasmas were characterized in this study, with 5 in genotype I (ntST 1, 2, 3, 6, 8), 4 in genotype II (ntST 4, 5, 10, 11), 1 in genotype III (ntST 7), and 1 in genotype IV (ntST 9) (Table 3, Fig. 2, Fig. 3).

**Table 3:** Genotypes, ntST, BLASTn results of bat-borne hemoplasmas based on 16S rRNA gene and their corresponding sample information of bats and ectoparasites.

Genotype	No. of sequenced samples	ntST	GenBank Accession number	Accession number of the closest match	Nucleotide identity (%)	Species	No. of sequences	Sampling site	Sampling year
I	53	1	ON426383	MK295627	99.1	*I. simplex*	1	Xianning	2021
						*M. davidii*	1	Xianning	2021
		2	ON426384		99.3	S*pinturnix* sp., *Eyndhovenia* sp.	1^‡^	Xianning	2021
		3	ON426385		98.3	*P. monoceros*	1	Xianning	2020
						*M. davidii*	2	Xianning	2021
		6	ON426388	MK295627	99.6	*M. adversus*	13	Xianning	2021
						*M. davidii*	1	Xianning	2018
							4	Jingzhou	2019
							9	Xianning	2021
						*M. fimbriatus*	1	Linyi	2015
						*E. serotinus*	1	Linyi	2015
						*P. monoceros*	1	Xianning	2020
							3	Xianning	2021
						S*pinturnix* sp., *Eyndhovenia* sp.	1^‡^	Xianning	2021
						S*pinturnix* sp., *Eyndhovenia* sp.	1^‡^	Xianning	2020
						*I. simplex*	1^‡^	Xianning	2021
		8	ON426390	MK295627	98.5	*M. davidii*	6	Xianning	2021
						*M. adversus*	1	Xianning	2020
							4	Xianning	2021
II	26	4	ON426386	KM538693	99.8	*M. davidii*	11	Xianning	2021
						*M. adversus*	8	Xianning	2021
		5	ON426387	KM538693	99.6	*M. adversus*	1	Xianning	2021
						*M. fimbriatus*	3	Linyi	2015
		10	ON426392	KM538698	99.6	*M. schreibersii*	2	Xianning	2021
		11	ON426393	KM538693	99.8	*M. pequinius*	1	Linyi	2015
III	4	7	ON426395	KY932691	95.0	*R. pusillus*	4	Xianning	2020
			ON426396					Xianning	2020
			ON426397					Xianning	2020
			ON426389					Xianning	2020
IV	2	9	ON426391	KY932722	95.5	*R. pusillus*	1	Xianning	2020
			ON426394			*M. davidii*	1	Xianning	2020
**Total**	**85**						**85**		

Note: ^‡^ Samples in pools.

The first clade including 53 sequences obtained in this study and was assigned as genotype I, which clustered together with hemoplasmas detected in *Myotis* bats from the Americas (Chile, Belize, and the United States). This genotype was detected in bats (*E. serotinus*, *M. adversus*, *M. davidii*, and *M. fimbriatus*), bat-flies (*P. monoceros*), bat-mites (S*pinturnix* sp., and *Eyndhovenia* sp.), and bat-ticks (*I. simplex*), and was identified in all sampling sites (Fig. 2). Hemoplasmas identified in 26 bats positioned in the second clade, namely genotype II, clustering within the human-pathogenic *Ca*. M. haemohominis group. This genotype was detected in *M. fimbriatus* and *M. pequinius* from Linyi, Shandong Province in 2015, and *M. adversus*, *M. davidii*, and *M. schreibersii* from Tongshan, Hubei Province in 2021 (Fig. 2). Four sequences exclusively from *Rhinolophus* bats formed a monophyletic clade, and were assigned as genotype III. The final clade comprising two sequences from *Rhinolophus pusillus* and *Myotis davidii*, positioned distant from other hemoplasma sequences (Fig. 2).

**Fig. 4:**
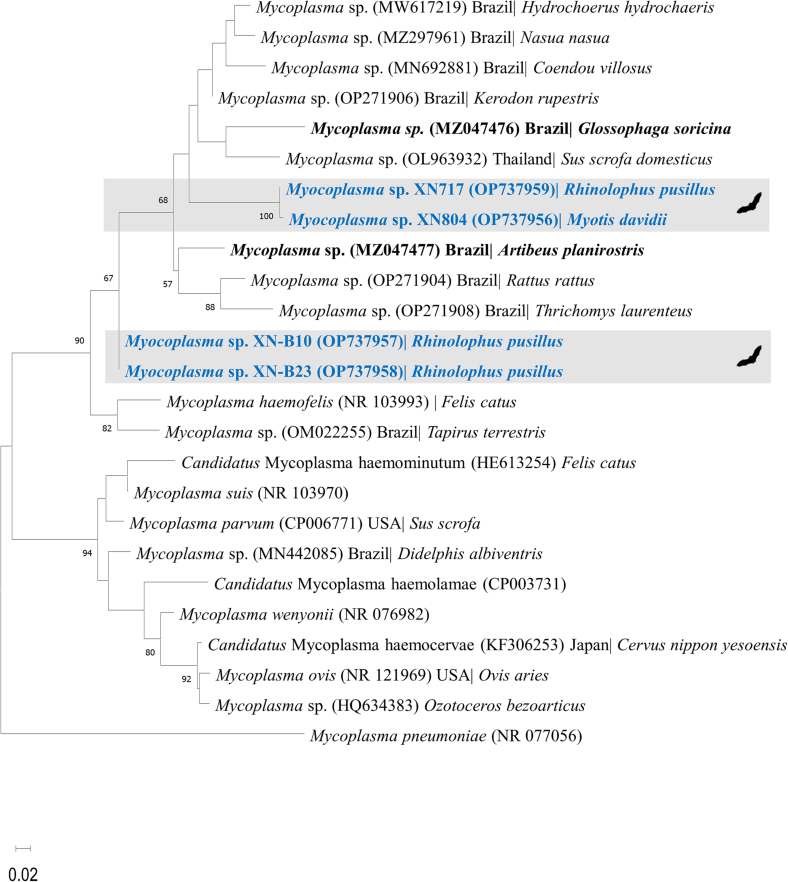
Phylogenetic analysis of hemoplasmas based on 23S rRNA gene (approximately 270 bp). The phylogenetic tree was constructed using the Maximum-likelihood method, Kimura 2-parameter model with Gamma distribution (K2 + G) and 1000 bootstrap analysis in MEGA**-**X. Sequences obtained from bats were shown in bold.

The two novel genotypes (III and IV) were further characterized with 23S rRNA gene phylogentic analysis. Phylogenetic tree of the 23S rRNA gene sequence (280 bp) showed that hemoplasmas were divided into 2 major clades (Fig. 4). The first clade, including two sequences from *R. pusillus* and *M. davidii*, positioned next to a group including a *Glossophaga soricina* bat (accession number MZ047476) in Brazil and other mammals. The second clade comprising 2 sequences from *R. pusillus*, clustered separately from other sequences within the Hemofelis group.

The hemoplasma DNA sequences obtained in this study were deposited in the GenBank with accession numbers ON426383–ON426397 and OP737956–OP737959.

## 4 Discussion

Our results demonstrated that bats from China carried abundant genetic diverse hemoplasmas. Hemoplasmas were identified in bats *M. fimbriatus*, *M. penquinius*, and *E. serotinus* in the family of Vespertilionidae, and *R. pusillus* in the family of Rhinolophidae for the first time, expanding our knowledge on the host range and geographic distribution of bat-related hemoplasmas.

### 4.1 Prevalence of hemoplasmas in bats

The overall prevalence of hemoplasmas in bats in this study is very high (18.0%, 103/572) but it still lower than those reports from Americas and Europe, which reported the hemoplasma infection rate in bats reaching to nearly 50–100% (43.0% (58/135) in Brazil [18], 51.0% (239/469) in Belize [15], 96.8% (30/31) in Spain [17]. The difference of hemoplasma PCR positive rate in this study and previous studies may be caused by different infection rate of hemoplasmas due to different locations and species of bats and genetic diversity of hemoplasmas affecting PCR sensitivity. Another possible reason is that tissue tropism of hemoplasmas. We have demonstrated the PCR positive rate of hemoplasmas was significantly high in blood samples than in liver and spleen tissues. This is a retrospective study and most samples of bats used were liver tissues. Therefore, our study may underestimate the hemoplasma prevalence in bats.

The prevalence of hemoplasmas was remarkably high in bats collected from Jingzhou, in 2019 (43.8% using liver) and Xianning, Hubei Province in 2021 (63.0% using blood and liver). A recent laboratory experiment revealed that *Mycoplasma haemomuris*-like bacterium infection in rodents reached peak infection level after 25–45 days post infection and stabilized in low bacterial loads for the rodents' lifetime [37]. Bats in the present study might share the same pattern of infection: bats with high frequency of infection, might be during an acute outbreak with high hemoplasma concentrations, while for bats in stable chronic stage of hemoplasmas, they may have a low bacterial load and leading to low PCR positive rate. This phenomenon was also observed in bats and other mammals in Brazil [18,38].

### 4.2 Genetic diversity and host specificity of hemoplasmas in bats

Bats and bat-ectoparasites harbored abundant genetic diverse hemoplasmas based on 16S rRNA gene. Genotype I, previously reported in bats from North and South America, was the major hemoplasmas identified in this study (Table 3). Genotype II, which positioned closely with human-pathogenic *Ca*. M. haemohominis clade, was identified in bats collected from the two provinces in China. Drs. Drancourt M. and Raoult D. proposed a sequence-based identification of a new bacterium that two bacteria are considered as two different species if their 16S rRNA genes share <97.0% similarity [39]. According to this criterion, genotype III and IV sharing 95.5% and 95.0% nucleotide similarity with other hemoplasma sequences in the GenBank, indicated they are novel genotypes. Phylogenetic analysis of 23S rRNA further characterized their novelty. Besides, Genotype III was exclusively identified in *R. pusilus* bats, while genotypes I and II were mainly found in bats belong to the genera of *Myotis* and *Miniopterus*. Host specificity was also observed in bats from Belize and Brazil, suggesting that hemoplasma evolution might be linked to bat speciation [15,18]. Inter-species transmission of hemoplasmas could happen among bats. A study in Nigeria found that one genotype was associated with both *Eidolon* and *Rhinolophus* bats [40]. Here, genotype IV was identified in both *Myotis* and *Rhinolophus* bats. Future studies are needed to elucidate the evolution of hemoplasmas in bats.

### 4.3 Public health significance of bat hemoplasmas

*Ca*. M. haemohominis infection in humans usually led to serious outcomes. The first human case of *Ca*. M. haemohominis was reported in 2011 in the UK [26]. Subsequently, human cases were also reported in New Caledonia, Australia, and Japan [16,24,25] (Fig. 2). It is reported that this pathogen has also been identified in bats in the family of Miniopteridae from Spain, Pteropondidae in New Caledonia, Pteropondidae and Molossidae in Nigeria [16,17,40]. Especially in New Caledonia, researchers found that *Ca*. M. haemohominis strains identified in patients, local flying foxes, and parasitic bat-flies were highly similar [16], indicating zoonotic potential from bats, and that bats might be natural hosts of *Ca*. M. haemohominis. We herein identified bats in the family of Vespertilionidae and Miniopteridae carried hemoplasmas which closely related to the human-pathogenic *Ca*. M haemohominis in China, and local physicians and officials should be alert to the potential emergence of human infections.

### 4.4 Transmission routes of hemoplasmas in bats

The transmission mechanism of hemoplasmas among animals remained unclarified, and direct contact, arthropod borne, airborne, and vertical transmission were postulated [6]. Bats are infested by a variety of ectoparasites, such as bat-flies, bat-mites, and bat-ticks. As a blood-borne pathogen, blood-sucking arthropods might play an important role in the transmission of hemoplasmas among bats. Previous studies reported the identification of hemoplasmas in bat-flies [16,18], and bat-ticks [41]. In this study hemoplasmas were identified in bat-flies (Nycteribiidae: *P. monoceros*, and *Nycteribia* sp.), bat-mites (Spinturnicidae: *Spinturnix* sp., and *Eyndhovenia* sp.), and bat-ticks (Ixodidae: *I. simplex*). These results indicated that bat-ectoparasites may play a role in transmission of hemoplasmas to bats, and bat-ectoparasites together with their hosts may be important in the maintenance and transmission of hemoplasmas in natural foci. The limitation of our study is that we could not demonstrate whether the hemoplasmas were the same between the host and their ectoparasites because we did not register the host-ectoparasite relationships. The role of ectoparasites as the vector and reservoir of hemoplasmas need to be confirmed by transmission experiments of hemoplasmas in laboratory animals.

Previous studies revealed that tick and flea might play a less important role in the transmission of hemoplasmas in rodents, dogs, and grey foxes [37,42,43]. An experiment found that none of the recipient rodents became infected after infested by fleas removed from hemoplasma-positive rodent donors, suggesting that flea-borne transmission might not play an important role in the transmission of hemoplasmas in rodents [37]. No strong association was found between hemoplasma infection status in dogs and the presence of parasitic ticks [42], or between hemoplasma prevalence in grey foxes and fleas [43]. A study in Brazil found that parasitic ticks and fleas from hemoplasma infected wild animals were negative [44].

Erythrocytic pathogens might be transmitted vertically from mother to baby during pregnancy and delivery. Previous study found that the prevalence and bacterial loads of *M. suis* in newborn but pre-suckling piglets were higher than sows, indicating the existence of vertical transmission [45]. Vertical transmission of hemoplasmas was also reported in dogs, cria, and cattle [[46], [47], [48]]. However, a laboratory infection experiment did not observe the evidence of vertical transmission in rodents [37]. In this study, fetuses of 11 hemoplasma-positive pregnant bats were negative for hemoplasmas, indicating that transplacental vertical transmission of hemoplasmas was unlikely to occur in bats. However, our result might be biased by the small sample size.

In conclusion, bats and bat-ectoparasites from China harbored abundant genetic diverse hemoplasmas including potential human-pathogenic hemoplasmas, indicating bats and bat-ectoparasites may play important roles in the maintenance and transmission of hemoplasmas in the natural foci.

## Authors' contributions

HJH, XJY, and RW designed the experiments. RW conducted the experiments with the help of ZML, QMP, and XLG. RW performed the result analysis. XX, CMZ, HJH, RW, ZML, and QMP carried out the field work. RW wrote the original manuscript. HJH and XJY revised the manuscript. All the authors read and approved the final manuscript.

## Fundings

This work was supported by the National Nature Science Funds of China (grant number: 81971939), the 10.13039/501100002858China Postdoctoral Science Foundation Funded Project (grant number: 2019M662720), and the Innovation Research of Hubei Postdoctoral Science and Technology.

## Declaration of Competing Interest

The authors declare no conflict of interest.

## Data Availability

Data will be made available on request.

## Appendix A Appendices

Supplementary Table S1 Primers used for species identification of bats and bat-ectoparasites and molecular detection of hemoplasmas. Supplementary Table S1

Supplementary Table S2 Comparison of the prevalence of hemoplasmas in blood, liver, and spleen samples of bats from Tongshan, Hubei Province, China, 2021. Supplementary Table S2
