# Supplementary material for: High prevalence and genetic diversity of hemoplasmas in bats and bat ectoparasites from China
Source: One Health. 2023 Feb 6;16:100498. doi: 10.1016/j.onehlt.2023.100498 (PMC9947411; doi:10.1016/j.onehlt.2023.100498)
Supplement: Supplementary Table S1 — Primers used for species identification of bats and bat-ectoparasites and molecular detection of hemoplasmas. [file mmc1.docx]

**Table S1.** Primers used for species identification of bats and their ectoparasites and molecular detection of hemoplasmas.

| Target species | Gene | Primers | Sequences (5' – 3') | Product length (bp) | References |
| --- | --- | --- | --- | --- | --- |
| Bat | *cytB* | cytB-F | CCATGAGGCCAAATATCCTTCTGAGG | 604 | [1] |
|  |  | cytB-R | TTGGCCAATGATAATGTAKGGRTGTTC |  |  |
| Bat fly | *COI* | LCO1490 | GGTCAACAAATCATAAAGATATTGG | 658 | [2] |
|  |  | HCO2198 | TAAACTTCAGGGTGACCAAAAAATCA |  |  |
| Bat mite | 16S | 16s+1 | CCGGTCTGAACTCAGATCAAGT | 543 | [3] |
|  |  | 16s-1 | GCTCAATGATTTTTTAAATTGCTGT |  |  |
| Bat tick | *COI* | LCO1490 | GGTCAACAAATCATAAAGATATTGG | 658 | [2] |
|  |  | HCO2198 | TAAACTTCAGGGTGACCAAAAAATCA |  |  |
| Hemotropic mycoplasmas | 16S | Myco 322s | GCCCATATTCCTACGGGAAGCAGCAGT | 600 | [4] |
|  |  | Myco 938as | CTCCACCACTTGTTCAGGTCCCCGTC |  |  |
|  |  | HemMycop16S-41s | GYATGCMTAAYACATGCAAGTCGARCG | 800 | [5] |
|  |  | HemMycop16S-938as | CTCCACCACTTGTTCAGGTCCCCGTC |  |  |
|  |  | HemMycop16S-322s | GCCCATATTCCTACGGGAAGCAGCAGT | 1000 |  |
|  |  | HemMycop16S-1420as | GTTTGACGGGCGGTGTGTACAAGACC |  |  |
|  | 23S | 23S-F | CGGGGCTAAGCTAAATACC | 300 | This study |
|  |  | 23S-R | TGCAAGAATATTAACTCGCTG |  |  |

Abbreviation: bp, base pair; *cyt*B, mitochondrial cytochrome B; *COI*, cytochrome oxidase subunit I.

**References**

[1] A. Ishii, K. Ueno, Y. Orba, M. Sasaki, L. Moonga, B. M. Hang'ombe, A. S. Mweene, T. Umemura, K. Ito, W. W. Hall, et al., A nairovirus isolated from African bats causes haemorrhagic gastroenteritis and severe hepatic disease in mice, Nat. Commun. 5 (2014). <http://doi.org/ARTN> 565110.1038/ncomms6651

[2] O. Folmer, M. Black, W. Hoeh, R. Lutz and R. Vrijenhoek, DNA primers for amplification of mitochondrial cytochrome c oxidase subunit I from diverse metazoan invertebrates, Mol. Mar. Biol. Biotechnol. 3 (5) (1994) 294-299.

[3] N. Bruyndonckx, S. Dubey, M. Ruedi and P. Christe, Molecular cophylogenetic relationships between European bats and their ectoparasitic mites (Acari, Spinturnicidae), Mol. Phylogenet. Evol. 51 (2) (2009) 227-237. <http://doi.org/10.1016/j.ympev.2009.02.005>

[4] M. Varanat, R. G. Maggi, K. E. Linder and E. B. Breitschwerdt, Molecular prevalence of *Bartonella*, *Babesia*, and hemotropic *Mycoplasma* sp. in dogs with splenic disease, J. Vet. Intern. Med. 25 (6) (2011) 1284-1291. <http://doi.org/10.1111/j.1939-1676.2011.00811.x>

[5] R. G. Maggi, M. C. Chitwood, S. Kennedy-Stoskopf and C. S. DePerno, Novel hemotropic Mycoplasma species in white-tailed deer (*Odocoileus virginianus*), Comp. Immunol. Microbiol. Infect. Dis. 36 (6) (2013) 607-611. <http://doi.org/10.1016/j.cimid.2013.08.001>
