# Supplementary material for: High prevalence and genetic diversity of hemoplasmas in bats and bat ectoparasites from China
Source: One Health. 2023 Feb 6;16:100498. doi: 10.1016/j.onehlt.2023.100498 (PMC9947411; doi:10.1016/j.onehlt.2023.100498)
Supplement: Supplementary Table S2 — Comparison of the prevalence of hemoplasmas in blood, liver, and spleen samples of bats from Tongshan, Hubei Province, China, 2021. [file mmc2.docx]

Table S2. Comparison of the prevalence of hemoplasmas in blood, liver, and spleen samples of bats from Tongshan, Hubei Province, China, 2021.

| Tissue type | No. of samples | No. of positive samples (%) | *P* value |
| --- | --- | --- | --- |
| blood | 99 | 78 (78.8) | <0.01 |
| liver | 92 | 20 (21.7) |  |
| spleen | 88 | 2 (2.3) |  |
